# Hypofractionated radiotherapy after conservative surgery for breast cancer: analysis of acute and late toxicity

**DOI:** 10.1186/1748-717X-5-112

**Published:** 2010-11-23

**Authors:** Letizia Deantonio, Giuseppina Gambaro, Debora Beldì, Laura Masini, Sara Tunesi, Corrado Magnani, Marco Krengli

**Affiliations:** 1 Department of Radiotherapy, University Hospital Maggiore della Carità, Novara, Italy; 2 Department of Epidemiology and Biostatistics, University Hospital Maggiore della Carità, Novara, Italy

## Abstract

**Background:**

A variety of hypofractionated radiotherapy schedules has been proposed after breast conserving surgery in the attempt to shorten the overall treatment time. The aim of the present study is to assess acute and late toxicity of using daily fractionation of 2.25 Gy to a total dose of 45 Gy to the whole breast in a mono-institutional series.

**Methods:**

Eighty-five women with early breast cancer were assigned to receive 45 Gy followed by a boost to the tumour bed. Early and late toxicity were scored according to the Radiation Therapy Oncology Group criteria. For comparison, a group of 70 patients with similar characteristics and treated with conventional fractionation of 2 Gy to a total dose of 50 Gy in 25 fractions followed by a boost, was retrospectively selected.

**Results:**

Overall median treatment duration was 29 days for hypofractionated radiotherapy and 37 days for conventional radiotherapy. Early reactions were observed in 72/85 (85%) patients treated with hypofractionation and in 67/70 (96%) patients treated with conventional fractionation (p = 0.01). Late toxicity was observed in 8 patients (10%) in the hypofractionation group and in 10 patients (15%) in the conventional fractionation group, respectively (p = 0.4).

**Conclusions:**

The hypofractionated schedule delivering 45 Gy in 20 fractions shortened the overall treatment time by 1 week with a reduction of skin acute toxicity and no increase of late effects compared to the conventional fractionation. Our results support the implementation of hypofractionated schedules in clinical practice.

## Background

Radiotherapy (RT) reduces the risk of local relapse and breast cancer mortality [1] and is offered to nearly all patients after conservative surgery and to selected patients after mastectomy. The international standard RT regimen after breast conservative surgery for early breast cancer delivers 25 daily fractions of 2 Gy to a total dose of 50 Gy over 5 weeks followed by 5 fractions of 2 Gy as a boost to the tumour bed [2]. The high number of women with breast cancer, receiving postoperative RT, led to think that a shorter course of irradiation would result in improved quality of life for patients, in potentially better integration with systemic treatments and in reduced costs. Therefore, alternative schedules based on a lower total dose delivered in fewer, larger fractions (hypofractionation) were firstly introduced in Canada and the United Kingdom (UK) [3,4]. The Canadian randomised trial [3] tested 42.5 Gy in 16 fractions against 50 Gy in 25 fractions. Results suggested equivalence in terms of local control and breast cosmetic results for the 16-fractions regimen.

The two most recent randomized studies [5,6] were conducted by the START Trials in order to test the effects of radiotherapy schedules using fraction size larger than 2.0 Gy. The START Trial A tested two dose levels of a 13-fractions regimen delivered over 5 weeks and the START Trial B compared 40 Gy in 15 fractions of 2.67 Gy in 3 weeks with a control group of 50 Gy in 25 fractions of 2.0 Gy over 5 weeks. These studies seem to offer rates of late adverse effects and local-regional tumour relapse at least as favourable as the standard schedule.

The aim of the present study carried out in a mono-institutional clinical setting is to assess acute and late toxicity of hypofractionated radiotherapy after conservative surgery using a regimen of 2.25 Gy/fraction to a total dose of 45 Gy to the whole breast followed by a boost comparing the results with those of a similar group of patients treated with conventional fractionation schedule.

## Methods

### Patients

From January 2006 to January 2008, 85 patients with invasive carcinoma of the breast treated with conservative surgery and biopsy of sentinel lymph node or axillary lymph node dissection were prospectively treated with whole breast irradiation of 45 Gy in 20 fractions, 2.25 Gy/fraction, followed by 9 Gy in 3 fractions to the tumour bed as a boost dose. Eligibility criteria were: age ≥ 60 years, T ≤ 2 cm, negative surgical margins and no indication to lymph node RT (≤ 3 positive lymph-nodes). Patients with history of contralateral breast cancer, multifocal disease, serious non-malignant disease (e.g. cardiovascular or pulmonary), severe mental or physical disorders were excluded from the study. The initial work-up included chest radiogram, liver ultrasound, bone scan, full blood count, kidney and liver function tests. Written informed consent was obtained from patients before start treatment following the rules of our institution.

A second group of 70 patients with similar characteristics in terms of clinical history, staging and type of surgery was retrospectively selected from patients treated since 2005 with post-operative breast RT with conventional schedule of 50 Gy in 25 fraction, 2 Gy/fraction followed by 10 Gy in 5 fractions to the tumour bed as a boost dose.

### Radiotherapy

All patients underwent post-operative RT planned using the three-dimensional treatment planning system, Pinnacle (Philips, Eindhoven, The Netherlands). Computed Tomography (CT) images were obtained by helical CT (Prospeed, General Electric Medical Systems, Milwaukee, WI), covering the entire thoracic region from the apex of the lung to the diaphragm, with patients in treatment position. Target and non-target volumes were outlined according to the criteria of the International Commission of Radiation Units (ICRU) and Measurements Reports 50 and 62 [7,8]. The clinical target volume (CTV) was defined as the entire palpable breast tissue starting 5 mm below the skin. The planning target volume (PTV) was obtained by adding 10 mm margin to the CTV, except in the direction of the skin. Ipsilateral lung was automatically outlined from the apex to the base and the left ventricle was manually outlined in case of left sided cancer. The treatment technique consisted of two opposed tangential fields by using 6-18 MV photon beams. Radiation fields were appropriately customized by multileaf collimator when needed in order to spare the surrounding healthy tissues. The angle of the beams was adjusted to minimize the irradiation of lung parenchyma and left ventricle. Appropriate physical wedge compensation was used to ensure a uniform dose distribution throughout the target volume. The total dose prescribed to the ICRU point was 45 Gy, delivered with 2.25 Gy per fraction, 5 days a week. A boost dose of 9 Gy in 3 fractions was given using a 6-9 MeV electron field, depending on the depth of the original tumor site. Dose calculation with a grid of 3 mm was performed using the collapsed cone convolution algorithm of the treatment planning system, including the correction for tissue heterogeneity. For each patient, dose-volume histograms (DVHs) for target, lung and left ventricle for left-sided cancers were calculated. The same technique had been used for the patients treated with conventional radiotherapy to a total dose of 50 Gy, 2 Gy/fraction and a boost dose of 10 Gy in 5 fractions to the tumour bed.

Biologically effective dose (BED) was calculated assuming alpha/beta ratio equals to 10 Gy for early reactions, 3 Gy for late reactions. In the group assigned to receive 45 Gy to the whole breast, BED was 55 Gy for early effects, 78 Gy for late effects versus 60 Gy for early effects, 83 Gy for late effects in the group treated with 50 Gy.

Sequential chemotherapy (cyclophosphamide - methotrexate -5-fluorouracil, doxorubicin - cyclophosphamide, 5-fluorouracil - epirubicin - cyclophosphamide) and hormone therapy given concomitantly to radiotherapy (tamoxifen or aromatase inhibitors) were given in 20/85 patients (24%) in the hypofractionation group while 65/85 patients (76%) received hormone therapy alone. The patients of the group treated by conventional fractionation received sequential chemotherapy in 20/70 cases (28%) and hormone therapy concomitant to radiotherapy in 50/70 cases (71%).

Early and late toxicity were scored according to the Radiation Therapy Oncology Group/European Organization for Research and Treatment Cancer (RTOG/EORTC) criteria in both groups of patients [9].

### Statistical analysis

The chi-square test and Fisher's exact test were used to compare the two treatment groups.

The association of early and late toxicity with breast volume, maximum radiation dose and chemotherapy was analyzed in the two treatment groups using Chi-square test and logistic regression. The Chi-square test was used for comparing acute and late toxicity between the patients treated with hypofractionation and those treated with conventional RT and for comparing the frequency of breast volume < 500 cc and ≥ 500 cc in the two treatment groups. Logistic regression was used to adjust the effect on toxicity of covariates, such as breast volume, maximum dose and chemotherapy. Chi-square test for trend was used to compare acute toxicity between the patients treated with hypofractionation and those treated with conventional RT.

The t-test was used to compare breast volume between the patients treated with hypofractionation and those treated with conventional RT.

Survival curves were obtained to show the cumulative probability of experiencing adverse effects during 6 months follow-up interval. The actuarial occurrence of late toxicity was calculated by the Kaplan-Meier method and the two treatment groups were compared using the Log-rank test.

Cox's proportional hazards regression model was fitted in order to obtain the hazard ratio (HR) for hypofractionation adjusted by volume and chemotherapy. A *p *value of less than 0.05 was considered to be statistically significant. Statistical analysis was performed using the SAS package version 8.02 (SAS Institute, Inc, Cary, NC, USA).

## Results

Both treatment groups were comparable in terms of age, performance status, tumour stage, adjuvant chemotherapy and hormone treatment. Differences were observed for lymph node stage, breast volume and breast maximum dose (Table 1).

**Table 1:** Patients' baseline characteristics.

	Hypofractionation No.		Conventional fractionation No.		P
Age	≥ 60		≥ 60		0.688
Mean	71.9		71.6		
IQR	9		11		

KPS					0.742
100	34	40%	26	37%	
90	51	60%	44	63%	

T stage					0.054
1	70	82%	49	70%	
2	14	17%	21	30%	
3	1	1%	0	0%	

N stage					0.045
0	69	83%	47	67%	
1	16	17%	23	33%	

Histologic type					0.357
Ductal	66	77%	54	77%	
Lobular	13	15%	7	10%	
Others	6	8%	9	13%	

Histologic grade					0.781
1	17	20%	13	18%	
2	48	56%	37	54%	
3	20	24%	20	28%	

Chemotherapy					0.375
yes	19	22%	20	29%	
no	66	88%	50	71%	

Hormone therapy					0.457
yes	66	78%	50	71%	
no	19	22%	20	29%	

Breast volume					0.039
≥ 500 cc	24	28%	29	41%	
< 500 cc	61	72%	41	59%	

Breast maximum dose					0.0009
> 107% **	26	36%	40	65%	
≤ 107%	59	64%	30	35%	

Surgery					0.584
Quadrantectomy	84	99%	68	97%	
Tumorectomy	1	1%	2	7%	

IQR = interquartile range

KPS = Karnofsky performance status

T = tumor

N = lymph-node

** > 53.5 Gy for conventional fractionation and > 48.1 Gy for hypofractionation

P = chi-square test or Fisher's exact test

Median time from surgery to RT was 29 days for hypofractionated RT and 37 days for conventional RT. No patient interrupted the treatment.

The mean follow-up was 15.0 months (median12.6; 25^th ^quartile 7.8 and 75^th ^quartile 20.8 months) in the hypofractionation RT group and 28.6 months (median32.2; 25^th ^quartile 22.1 and 75^th ^quartile 40.0 months) in the conventional fractionation group.

### Acute toxicity

Early reactions, consisting in skin erythema, were observed in 72 patients (85%) in the hypofractionation group and in 67 (95%) in the conventional RT group (chi-square p = 0.01; chi-square test for trend p = 0.001) (Table 2).

**Table 2:** Acute radiation reactions (RTOG scale).

Grade	Hypofractionation		Conventional fractionation		p
	No	%	No	%	
0	13	16	3	4	< 0.001
1	51	60	34	49	
2	19	22	29	41	
3	2	2	4	6	

**Figure 1:**
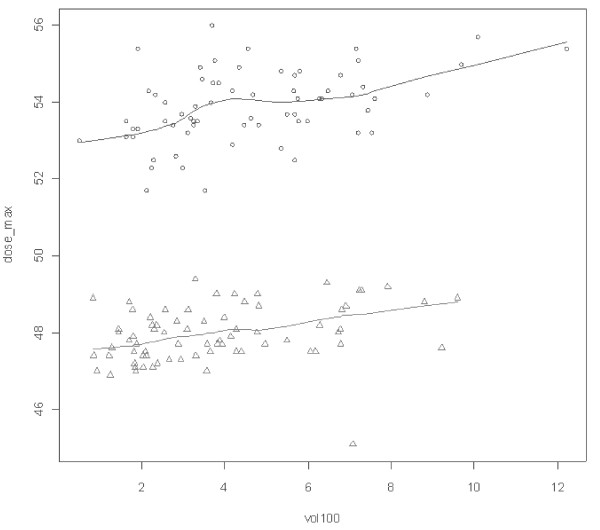
**The figure shows the relation between breast volume (expressed in cc × 100) and maximum dose (expressed in Gy)**. Standard fractionation is represented by circles and hypofractionation by triangles.

A significant correlation by chi-square test (p = 0.001) between breast volume and maximum dose was found for the occurrence of acute toxicity (Figure 1). Adjuvant treatments did not influence acute toxicity.

Logistic regression analysis was carried on for adjusting for potential confounders. In this analysis, acute skin toxicity was classified in two categories: mild (G0 and G1) and severe (G2 and G3). Hypofractionation reduced the risk of severe acute toxicity: odd ratio (OR) adjusted for volume was 0.45 (95% CI = 0.23-0.93). Including in the analyses also maximum breast dose and chemotherapy did not provide a significant contribution to the model fit.

### Late toxicity

**Figure 2:**
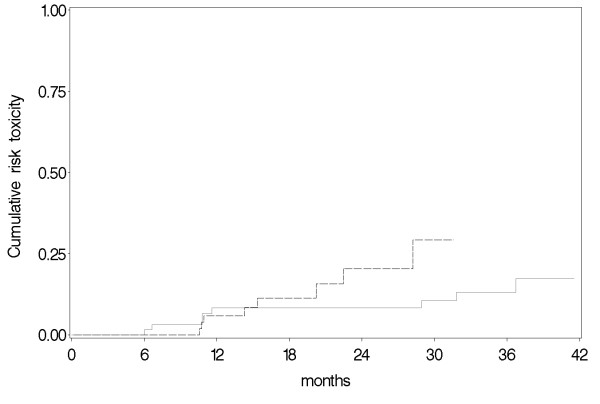
**Time course of breast fibrosis as a cumulative risk of late toxicity in patients treated with standard RT (continuous line) and patients treated with hypofractionation (dotted line)**.

Late toxicity was assessed after 6 months in 76/85 patients in the hypofractionation group and in 67/70 patients in the standard RT group since 9 and 3 patients, respectively, were lost at follow-up and were not included in the statistical analysis. Late toxicity according to the RTOG criteria was observed in 8 patients (10%) in the hypofractionation group and in 10 patients (15%) in the conventional fractionation group (Table 3). The difference was not statistically significant (chi-square p = 0.4). Cumulative occurrence of late toxicity over time was analyzed using Kaplan-Meier method and compared by log-rank test, resulting not statistically significant (p = 0.17) (Figure 2). At 12 and 30 months, the risk of late toxicity was 5.9% and 29.2% in the group treated by hypofractionation, 8.2% and 10.6% in the group treated by standard RT, respectively.

**Table 3:** Late radiation reactions (RTOG scale).

Grade	Hypofractionation		Conventional fractionation		p
	No	%	No	%	
0	68	90	57	85	0.4
1	8	10	10	15	
2	0	0	0	0	
3	0	0	0	0	

Cox's proportional hazards regression analysis showed that hypofractionation, adjusted by volume and chemotherapy, was not associated with the hazard of late toxicity (HR = 2.16; 95% CI = 0.68-6.84; p = 0.19). In the same model, we observed that breast volume increased the hazard of late toxicity over time (HR = 1.27; 95% CI = 1.04-1.55; p = 0.016).

## Discussion

Although a number of preliminary data support the use of partial breast irradiation in low risk patients [10], whole breast irradiation will probably remain the standard treatment for intermediate and high risk cases. Breast irradiation after conservative surgery is usually given daily for 5-6 weeks. Results of many trials showed that shorter fractionation schedules are as effective as the conventional schedule of 50 Gy in 25 fractions in terms of preventing recurrence of cancer in the breast [3-5].

The linear-quadratic model is typically used to calculate the biologically equivalent dose taking into account a larger dose per fraction over a shorter period of time [11]. As a matter of fact, the size of dose per fraction may influence the tolerance of normal tissues and also the therapeutic results [12].

This model predicts that the normal tissue toxicity is not increased when the fraction dose is modestly increased and the total dose is reduced accordingly to the linear-quadratic formalism [11].

In the present study women with more than 60 years were enrolled. In literature, some authors used age as a selection criteria [13] while other studies, like START trial, considered eligible women aged over 18 years.

As far as the incidence and the grade of acute skin toxicity are concerned, the present study showed a significant difference (p = 0.01) between the two treatment groups with lower toxicity in the hypofractionation group. A similar finding was reported in a Japanese retrospective study [14] in which the authors observed that acute toxicity by hypofractionated RT (40 Gy in 16 fractions, fraction size 2.5 Gy) was milder than that by the conventional schedule (50 Gy in 25 fractions, fraction size 2 Gy) (p = 0.01). Other literature studies reported substantially similar results. Whelan et al. [3] in a randomized trial of 1234 women with early stage negative nodes breast cancer, comparing conventional fractionation (50 Gy in 25 fractions) versus hypofractionation (42.5 Gy in 16 fractions) found no difference regarding the early toxicity between the two regimens. Substantially similar results were reported by Olivotto et al. [15] in a non randomized study on 186 patients treated with 44 Gy in 16 fractions over 22 days. The authors reported results to be comparable to their historical patients. No significant differences in acute skin reaction were showed also in an Egyptian study [13] analysing 30 patients randomized to receive adjuvant RT with conventional schedule or hypofractionation of 42.5 Gy in 16 fractions.

The present study showed a significant difference in volume size (p = 0.039) between the two patient groups and an association between severity of acute effects and breast volume (p = 0.001). In order to overcome confounding analyses were adjusted by breast volume. In this regard, the Egyptian study showed also a significant correlation between breast volume and severity of acute skin reactions. In the present study, systemic adjuvant treatment had not significant correlation with toxicity as reported also in other literature series [12].

The reduction of acute toxicity in patients treated with hypofractionated RT in the present series might be explained by the BED value of acute reaction calculated by linear quadratic model that was lower than that of the conventional schedule and by the breast volume that was larger in the group of conventional fractionation (p = 0.039). As a matter of fact, large volume breasts are frequently associated with dose inhomogeneity and this fact may influence the occurrence of acute reactions. In fact, the present study found that voluminous breasts had more frequently a maximum dose higher than the cut off of 48.1 and 53.5 Gy, i.e. the 107% of prescribed dose for hypofractionation and standard RT, respectively (Table 1).

Similarly to what reported in other literature studies, no statistical difference (p = 0.4) for late toxicity was found between patients treated by hypofractionation and those treated by conventional fractionation [9]. However, the UK randomised trial [4] on hypofractionated radiotherapy using fraction sizes of 2, 3 and 3.3 Gy in 1410 randomized patients found that the 3.3 Gy schedule to a total dose of 42.9 Gy allowed to obtained the worst cosmetic result meaning that late effects may worsen when the fraction size is largely increased. In our series, the actuarial occurrence of late toxicity between the two groups, by Kaplan-Meier, resulted not significant (p = 0.17). At 12 months and 30 months, the risk of late toxicity was 5.9% and 29.2% respectively in the group treated by hypofractionation, 8.7% and 10.6% in the group treated by standard RT (Figure 2). Similarly to what observed for acute toxicity's results, breast volume increased the risk of late toxicity (p = 0.016) when analysed with Cox's proportional hazards regression model.

Main limitations of the present study were the non-randomized design and consequently the possible presence of some minor bias such as the different breast size, the different percentage of patients with maximum dose higher than cut off in the two groups and the relatively shorter follow-up time in the hypofractionation group that could affect the incidence of late side effects. Nevertheless, statistical adjustment made possible to reach a conclusion in respect to the association of fractionation and adverse effects.

## Conclusions

The data reported in the present study confirm the feasibility of the hypofractionated RT with 2.25 Gy per fraction to a total dose of 45 Gy in patients with invasive breast cancer in daily practice. Patients well tolerated the treatment with excellent compliance and nobody stopped the radiotherapy course that lasted 8 days less than that of conventional fractionation.

Acute dermatitis by hypofractionation was milder than that by the conventional RT (p = 0.01). No significant difference of late effects (p = 0.4) compared to the conventional schedule was found. These results, like those from other literature studies, support the implementation of hypofractionated radiation schedules in clinical practice.

## Competing interests

The authors declare that they have no competing interests.

## Authors' contributions

LD was the study coordinator, participated in the development of the study and drafted the manuscript. CM and ST worked on analysis of data, GG, DB and LM participated in the design of the study and were involved in continuing optimization. MK was the study chairman, developed the design of the study, was involved in continuing optimization and helped to draft the manuscript. All authors read and approved the final manuscript.
